# Mucus Plugging as a Treatable Trait Across the Asthma–COPD Spectrum: The Role of Type 2 Cytokine Blockade and Quantitative Imaging

**DOI:** 10.3390/biomedicines14040891

**Published:** 2026-04-14

**Authors:** Pier-Valerio Mari, Alberto Ricci, Angelo Coppola, Davide Onofrio Fontana, David Selvaggio, Lorenzo Carriera, Simone Ielo, Matteo Siciliano, Loreta Di Michele, Veronica Ojetti

**Affiliations:** 1 Internal Medicine, San Carlo di Nancy Hospital, Via Aurelia 275, 00165 Rome, Italy; vojetti@gvmnet.it; 2 Division of Pneumology, Department of Clinical and Molecular Medicine, Sapienza University of Rome, AOU Sant’Andrea, 00189 Rome, Italy; alberto.ricci@uniroma1.it; 3 UOC Pneumologia, Ospedale San Filippo Neri-ASL Roma 1, 00135 Rome, Italy; coppolangelo@gmail.com; 4 Department of Pulmonology, UniCamillus International Medical University of Rome, 00131 Rome, Italy; 5 Internal Medicine, Fondazione Policlinico Universitario Campus Bio-Medico, 00128 Rome, Italy; d.fontana@policlinicocampus.it; 6 UOS di Malattie dell’Apparato Respiratorio Ospedale Cristo Re, 00167 Roma, Italy; davselv@yahoo.it; 7 Faculty of Medicine and Surgery, Università Cattolica del Sacro Cuore, 00168 Rome, Italy; 8 Department of Pulmonology and Sub-Intensive Respiratory Unit, Santa Maria della Misericordia Hospital, 06156 Perugia, Italy; 9 Pulmonology and Respiratory Intensive Care Unit, Ospedale San Donato, USL Toscana Sud-Est, 52100 Arezzo, Italy; simone.ielo@uslsudest.toscana.it; 10 Independent Researcher, 00100 Rome, Italy; mat.sic89@gmail.com; 11 San Camillo-Forlanini Hospital, 00152 Rome, Italy; dottoressadimichele@gmail.com

**Keywords:** mucus plugs, treatable traits, type 2 inflammation, biologics, dupilumab, tezepelumab, quantitative CT, asthma, COPD, precision medicine

## Abstract

**Background:** Airway mucus plugging is a key but long-overlooked mechanism of persistent airflow obstruction in both asthma and chronic obstructive pulmonary disease (COPD). Type 2 (T2) cytokines, particularly interleukin (IL)-4 and IL-13, drive goblet cell metaplasia, MUC5AC overexpression, and impaired mucociliary clearance, while eosinophil-derived products increase mucus viscosity and promote plug persistence. **Methods:** A comprehensive narrative review was conducted by searching PubMed and ClinicalTrials.gov databases from inception to February 2026. Search terms included “mucus plugs,” “mucus plugging,” “biologics,” “dupilumab,” “tezepelumab,” “mepolizumab,” “benralizumab,” “IL-4,” “IL-13,” “MUC5AC,” “quantitative CT,” “functional respiratory imaging,” “asthma,” and “COPD.” Studies were included if they reported original data or systematic evidence on mucus plug quantification, biologic-mediated changes in mucus plug scores, or imaging modalities for mucus assessment in asthma or COPD. Editorials, case reports with fewer than three patients, and studies not available in English were excluded. Two authors (P.-V.M. and A.C.) independently screened titles and abstracts; discrepancies were resolved by consensus. Randomized controlled trials, observational studies, and preclinical studies evaluating mucus plug outcomes and T2-targeted therapies were included. Reference lists of retrieved articles were hand-searched for additional relevant publications. **Results:** A recent systematic review identified multiple randomized controlled trials and observational studies that showed CT-assessed mucus plug scores go down with biologic therapies targeting the T2 pathway in asthma. Observational data extend this evidence to anti-IL-5/IL-5Rα agents. The VESTIGE trial provided the first functional respiratory imaging evidence of mucus plug resolution with dupilumab. In COPD, the BOREAS/NOTUS and MATINEE trials established the efficacy of dupilumab and mepolizumab in eosinophilic phenotypes; however, differences in inclusion criteria—particularly regarding FeNO thresholds and prior exacerbation burden—may explain divergent effects on lung function endpoints. Mucus plug outcomes have not been evaluated in COPD biologic trials. Quantitative imaging modalities, including HRCT mucus plug scoring, functional respiratory imaging, and hyperpolarized gas MRI, now enable objective assessment of mucus burden. **Conclusions:** Mucus plugging meets the definition of a treatable trait: it can be measured with CT scoring, it matters clinically, and it responds to T2 cytokine blockade. Adding mucus plug assessment to routine clinical evaluation, together with mucolytic strategies where needed, could move treatment decisions from empirical to biology-based across the asthma–COPD spectrum. Further studies are needed to confirm that mucus plug scoring works as a biomarker of treatment response in COPD and to test whether combining biologics with mucolytics improves outcomes.

## 1. Introduction

Chronic airway diseases—asthma and COPD together—affect over 500 million individuals worldwide, yet a striking proportion remain symptomatic despite therapeutic advances [1,2]. The traditional approach, in which a disease label dictates treatment, is giving way to a precision medicine model centered on treatable traits: measurable characteristics that are clinically relevant and amenable to targeted intervention [3].

Airway mucus plugging is one such trait—pivotal, yet historically underrecognized [4,5]. Dunican and colleagues used a bronchopulmonary segment-based CT scoring system in the Severe Asthma Research Program (SARP) and found mucus plugs in 58% of patients with asthma; strikingly, the plugs persisted in the same airway segments for years [4,5]. Longitudinal SARP-3 data then showed that changes in mucus plug scores correlate inversely with changes in FEV_1_, supporting a causal link between plug burden and airflow obstruction [6].

At its core, pathologic mucus production is driven by type 2 (T2) inflammation. IL-13, acting through the IL-4Rα/IL-13Rα1 signaling complex, triggers goblet cell metaplasia, MUC5AC mucin overexpression, and impaired mucociliary clearance [7,8]. But the problem does not end there. Eosinophil peroxidase (EPO) generates reactive oxidants that crosslink cysteine thiol groups in mucin polymers, stiffening the gel and promoting plug formation [9]. Charcot–Leyden crystals—composed of galectin-10 (Gal10) released from degranulating eosinophils—compound the issue by acting as T2 adjuvants and increasing mucus tenacity [10].

Can mucus plugs be dissolved by blocking the cytokines that produce them? Recent trial data suggest they can. A systematic review by Aegerter and colleagues documented that biologic therapies consistently reduce CT-assessed mucus plug scores in severe asthma, with the strongest evidence from the VESTIGE trial (dupilumab) and the CASCADE trial (tezepelumab) [11,12,13]. Observational studies extend these findings to anti-IL-5/IL-5Rα agents and omalizumab [14]. In COPD, the approval of dupilumab following the BOREAS and NOTUS trials [15] and the MATINEE trial of mepolizumab [16] have opened a new therapeutic frontier—but whether these agents also clear mucus plugs in COPD remains entirely unexplored.

The present review has three objectives: first, to synthesize the pathobiological rationale for treating mucus plugging as a treatable trait across the asthma–COPD spectrum; second, to evaluate the evidence from biologic trials with emphasis on quantitative imaging outcomes; and third, to propose a practical framework for integrating mucus plug assessment into clinical decision-making.

## 2. Pathobiology of Mucus Plugging in T2 Inflammation

### 2.1. The IL-4/IL-13 Axis and Goblet Cell Metaplasia

**Figure 1:**
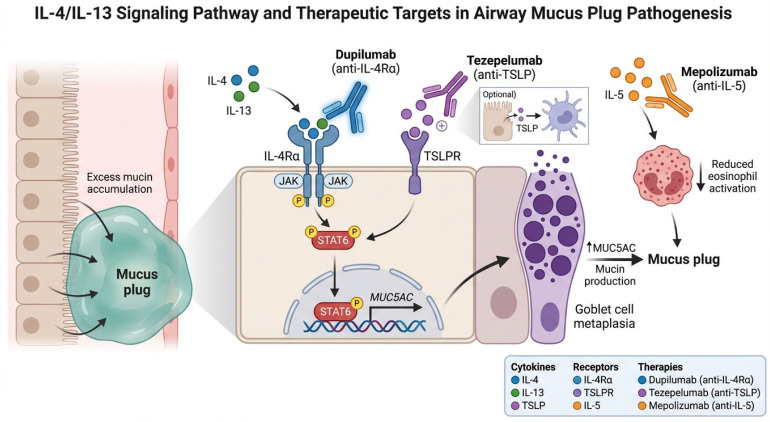
Schematic representation of the IL-4/IL-13 signaling pathway and its effects on airway epithelial biology, goblet cell metaplasia, and mucin production. The figure illustrates the molecular targets of dupilumab (IL-4Rα), tezepelumab (TSLP), and anti-IL-5 agents in relation to mucus plug pathogenesis.

The T2 cytokines IL-4 and IL-13 exert overlapping but distinct effects on airway epithelial biology. IL-13 is the principal inducer of goblet cell metaplasia via upregulation of the transcription factor SPDEF and concomitant suppression of FOXA2, a master regulator of airway epithelial homeostasis [17,18]. This transcriptional switch drives the conversion of ciliated cells to MUC5AC-secreting goblet cells, resulting in a marked shift in the MUC5AC-to-MUC5B ratio that alters mucus viscoelastic properties [19]. These interconnected pathways—from IL-13-driven goblet cell metaplasia to eosinophil-mediated mucin crosslinking and mucociliary clearance impairment—are summarized schematically in Figure 1. Bonser and colleagues demonstrated that MUC5AC-rich mucus becomes tethered to the airway epithelium, physically impeding mucociliary transport even when ciliary function is preserved [20], a finding consistent with the established role of mucin polymer structure in innate airway defense [21]. IL-4 contributes primarily through its role in Th2 cell differentiation, IgE class-switching, and VCAM-1 upregulation, which facilitates eosinophil adhesion and transmigration into the airway.

Of particular interest, corticosteroid treatment partially reverses IL-13-induced goblet cell metaplasia by restoring FOXA2 expression and suppressing SPDEF [18]. This observation provides a mechanistic rationale for the residual mucus plug burden observed in patients on high-dose inhaled corticosteroids (ICS): while ICS attenuate T2 inflammation, they do not fully normalize mucin gene expression, particularly when upstream T2 cytokine production is sustained. Dupilumab, by blocking both IL-4 and IL-13 signaling through the shared IL-4Rα subunit, addresses this limitation at the receptor level. From a clinical standpoint, this means that patients with persistent mucus plugging despite high-dose ICS may benefit from dupilumab, which addresses IL-13-driven mucin overproduction at the receptor level rather than downstream.

### 2.2. Eosinophil-Derived Products and Mucus Gel Stiffening

Eosinophils contribute to mucus plug pathology through multiple mechanisms. EPO catalyzes the oxidation of thiocyanate and bromide in the presence of hydrogen peroxide, generating reactive oxidants that crosslink cysteine thiol groups in mucin polymers [4]. This thiol-mediated crosslinking stiffens the mucus gel, rendering it resistant to mucociliary clearance and expectoration. Persistent sputum EPX levels, a protein biomarker of eosinophilic degranulation, correlate with high mucus plug scores and severe airflow obstruction in the SARP-3 cohort, even in patients receiving anti-IL-5 therapy.

**Figure 2:**
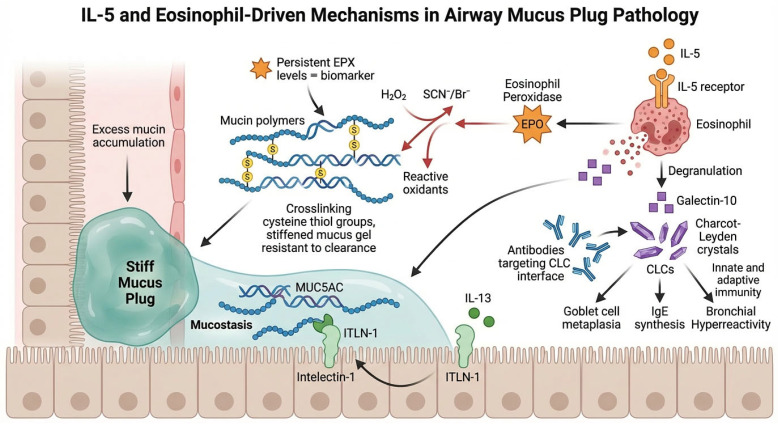
This schematic illustrates how IL-5-driven eosinophil activation contributes to airway mucus plug pathology via the release of cytotoxic proteins (EPO, EPX), the formation of Charcot–Leyden crystals from galectin-10, and their combined effects on mucin crosslinking, immune activation, and impaired mucociliary clearance.

Charcot–Leyden crystals (CLCs), formed by spontaneous crystallization of galectin-10 released from degranulating eosinophils, represent an additional pathogenic factor [10]. Persson and colleagues demonstrated that crystalline Gal10 acts as a potent T2 adjuvant, stimulating innate and adaptive immunity, goblet cell metaplasia, IgE synthesis, and bronchial hyperreactivity in a humanized mouse model [10]. Antibodies targeting the CLC crystallization interface dissolved pre-existing crystals in patient-derived mucus within hours, reversing crystal-driven inflammation. The identification of intelectin-1 (ITLN-1) as an IL-13-induced, MUC5AC-binding protein that promotes mucostasis has further expanded this mechanistic framework [22]. These mechanisms are illustrated in Figure 2. In practice, persistent sputum EPX elevation in patients on anti-IL-5 therapy may signal ongoing mucus crosslinking that requires a complementary therapeutic approach.

### 2.3. Mucociliary Clearance Impairment

T2 inflammation impairs mucociliary clearance (MCC) in a dose-dependent fashion. Corcoran and colleagues used radiolabeled particle scintigraphy to demonstrate that increasing T2 biomarker levels (FeNO, CCL26, NOS2) are associated with progressively decreasing MCC rates in patients with asthma [23]. The combination of MUC5AC overproduction, mucin crosslinking, and reduced ciliary function creates a self-reinforcing cycle: retained mucus promotes eosinophil accumulation, which in turn generates further oxidative crosslinking and crystal deposition. This vicious cycle provides a strong rationale for upstream cytokine blockade as a strategy to break the pathogenic loop. Clinically, these findings suggest that FeNO and mucociliary clearance assessment could help identify patients trapped in this self-reinforcing cycle who are most likely to benefit from upstream cytokine blockade.

### 2.4. The Microbiome Dimension

Retained mucus plugs may also serve as a reservoir for microbial colonization. Tanabe and Matsumoto recently reviewed the interplay between airway microbiome composition, inflammatory milieu, and mucus plugging in chronic airway disease [24]. This microbial–mucus interaction may partially explain the persistence of mucus plugs despite biologic therapy and raises the question of whether adjunctive mucolytic or antimicrobial strategies could enhance treatment response.

## 3. Quantitative Imaging of Mucus Plugs

### 3.1. CT-Based Mucus Plug Scoring

The CT-based mucus plug scoring system developed by Dunican and colleagues evaluates each of 18 to 20 bronchopulmonary segments for the presence of intraluminal high-attenuation opacities indicative of mucus plugging [4]. A mucus plug score (MPS) of 0 to 20 is generated, with a score of 4 or greater defining the “high mucus phenotype.” This approach has been adopted as the standard imaging endpoint in biologic trials and has demonstrated associations with airflow obstruction (FEV_1_), air trapping, exacerbation frequency, and eosinophilic biomarkers [25].

Longitudinal data from SARP-3 have established that mucus plugs are persistent at both the patient and the segment level, with participants having baseline plugs being 2.8 times more likely to have plugs at 3-year follow-up [6]. The change in MPS over time correlates significantly and negatively with change in FEV_1_ (ρ = −0.35; *p* < 0.001), providing evidence that mucus plug resolution translates into functional improvement. Ultra-high-resolution CT, as employed by Oga and colleagues, has further improved plug detection sensitivity, demonstrating differential associations between T2 biomarkers and mucus plugging across the asthma, asthma–COPD overlap (ACO), and COPD spectrum [26].

### 3.2. Functional Respiratory Imaging (FRI)

FRI, a computational fluid dynamics-based technology applied to paired inspiratory/expiratory HRCT scans, quantifies specific regional airway volumes (siVaw) and airway resistance at the lobar and segmental level. The VESTIGE trial represented the first application of FRI to assess biologic-mediated mucus plug reduction. In this phase 4 RCT, dupilumab led to a numerical but not statistically significant increase in siVaw at total lung capacity (LS mean difference vs. placebo: +21.8%, 95% CI −7.7 to 51.3; *p* = 0.14), alongside significant reduction in FeNO and mucus plug scores [12]. FRI uniquely allows regional mapping of ventilation changes attributable to plug resolution, a capability not available with spirometry alone.

### 3.3. Hyperpolarized Gas MRI

Hyperpolarized helium-3 (^3^He) MRI provides direct visualization of ventilation defects at the bronchopulmonary segment level. In a secondary analysis of SARP-3, Mummy and colleagues demonstrated that segments containing mucus plugs had a median ventilation defect percentage (VDP) of 25.9%, compared with 1.4% in plug-free segments (*p* < 0.001) [27]. This strong spatial concordance between CT-identified mucus plugs and MRI-defined ventilation defects supports the clinical relevance of the mucus plug phenotype and establishes a mechanistic link between plug burden and regional ventilation impairment.

### 3.4. Quantitative CT Disease Probability Mapping

Disease probability maps (DPMs) from paired inspiratory–expiratory CT registration, enable quantification of functional small airway disease (fSAD), air trapping, and regional lung expansion [28,29]. In the SARP cohort, fSAD was highest in the most severe asthma phenotype (cluster 5: 38.8%), and lung segments with air trapping more frequently had airway mucus plugging than segments without (48% vs. 18%; *p* ≤ 0.0001). These parametric imaging endpoints offer potential as treatment-responsive biomarkers in future biologic trials.

## 4. Biologic Therapies and Mucus Plug Reduction

### 4.1. Dupilumab

Dupilumab, a fully human monoclonal antibody that blocks IL-4Rα and thereby inhibits both IL-4 and IL-13 signaling [30,31], has the most robust evidence base for mucus plug reduction. In the systematic review by Aegerter et al. [11], dupilumab consistently demonstrated significant reductions in CT mucus plug scores across multiple studies in patients with T2-high severe asthma. The VESTIGE trial (phase 4, N = 109, BEC ≥ 300 cells/μL and FeNO ≥ 25 ppb) extended these findings by demonstrating that dupilumab reduced airway inflammation and mucus plugging while improving airway volumes and flow on FRI [12]. At week 24, 57% of dupilumab-treated patients achieved FeNO below 25 ppb, compared with 11% in the placebo group (OR 9.8, 95% CI 3.1–30.8; *p* < 0.001).

A post hoc analysis of the VESTIGE trial further characterized dupilumab’s effect on mucus burden [32]. At baseline, 67.2% of dupilumab-treated patients had a high mucus plug score (≥4); by week 24, this proportion had decreased to 32.8%, whereas it remained essentially unchanged in the placebo group (73.3% to 76.7%). The greatest improvements in pre-bronchodilator FEV_1_ were observed in the high-mucus-plug score subgroup (LS mean difference vs. placebo: 16.77 percentage points, 95% CI 9.81–23.73; *p* < 0.0001), and moderate-to-strong correlations between changes in mucus plug score and changes in FEV_1_ were documented (r = −0.61; *p* < 0.0001). These findings provide compelling evidence that mucus plug resolution contributes directly to the lung function gains observed with dupilumab. An important caveat, however, is that approximately one-third of dupilumab-treated patients remained in the high mucus plug score category, and most still had detectable plugs after 24 weeks of treatment. As Fahy highlighted in an accompanying editorial [33], these residual plugs underscore the need for a more comprehensive treatment approach—one that combines anti-inflammatory biologics with muco-active strategies to restore airway patency, analogous to the multifaceted management of occlusive vascular clots in cardiovascular disease.

Dupilumab’s effect rationale on mucus plugs is tricky: by blocking IL-13 signaling, dupilumab directly addresses the transcriptional program driving goblet cell metaplasia and MUC5AC overproduction. By reducing eosinophilic infiltration (through IL-4-mediated VCAM-1 downregulation), it may also attenuate the EPO-driven crosslinking that stiffens the mucus gel.

### 4.2. Tezepelumab

Tezepelumab, a human anti-TSLP monoclonal antibody, acts upstream of T2 cytokine production by blocking thymic stromal lymphopoietin, an epithelial alarmin. In the CASCADE trial (exploratory phase 2, N = 82), tezepelumab reduced CT mucus plug scores by a mean of −1.7 ± 2.6 points versus no change in the placebo group [13]. Baseline mucus plug scores correlated positively with blood eosinophils, eosinophil-derived neurotoxin, FeNO, IL-5, and IL-13, and negatively with FEV_1_ and forced mid-expiratory flow. Reductions in mucus plug scores were correlated with improvements in lung function and reductions in blood eosinophil counts, establishing a link between upstream alarmin blockade and downstream mucus clearance.

The broader anti-inflammatory spectrum of tezepelumab—which inhibits not only the IL-4/IL-13 axis but also IL-5-driven eosinophilia and ILC2 activation—may be particularly relevant for patients with mixed or non-eosinophilic mucus plugging phenotypes. In the CASCADE analysis, greater mucus plug reductions were observed in patients with T2-high biomarker profiles, yet effects were also detectable across the wider population [14].

### 4.3. Anti-IL-5/IL-5Rα Agents

Mepolizumab (anti-IL-5), benralizumab (anti-IL-5Rα), and reslizumab (anti-IL-5) deplete or suppress eosinophils but do not directly block IL-13-mediated goblet cell metaplasia. Observational studies without placebo control have demonstrated reductions in mucus plug scores following treatment with these agents [34], particularly in patients with high baseline eosinophilia [14]. In a prospective observational study of 47 patients with severe eosinophilic asthma, Campisi and colleagues showed that 12 months of mepolizumab therapy reduced the median mucus plug score from 4 to 1 (*p* < 0.0001), with reductions correlating significantly with decreases in blood eosinophils (r = 0.40), sputum eosinophils (r = 0.58), and oral corticosteroid dose (r = 0.38), as well as with improvements in FEV_1_ (r = −0.37) [35]. Götschke and colleagues extended these observations to a broader range of biologics in a retrospective analysis of 113 biologic-naïve patients with severe asthma [36]. Mucus plugging was present in 77% of patients at baseline (median MPS = 4), and the baseline MPS independently predicted improvement in FEV_1_ (β = 0.72; *p* = 0.01) and Asthma Control Test score (β = 0.24; *p* = 0.001) after 4 months of biologic therapy, irrespective of the specific agent used. These data support mucus plug scoring as a predictive biomarker of treatment response that may complement conventional type 2 biomarkers. Tang and colleagues found that mepolizumab normalized serum EPX in 96% of patients but sputum EPX in only 49%, and lung function remained abnormal even when sputum EPX normalized. This analysis suggests that eosinophil depletion alone may be insufficient to resolve established mucus plugs, consistent with the concept that mucin overproduction (IL-13-driven) and mucus crosslinking (EPO-driven) are partially independent pathways.

**Figure 3:**
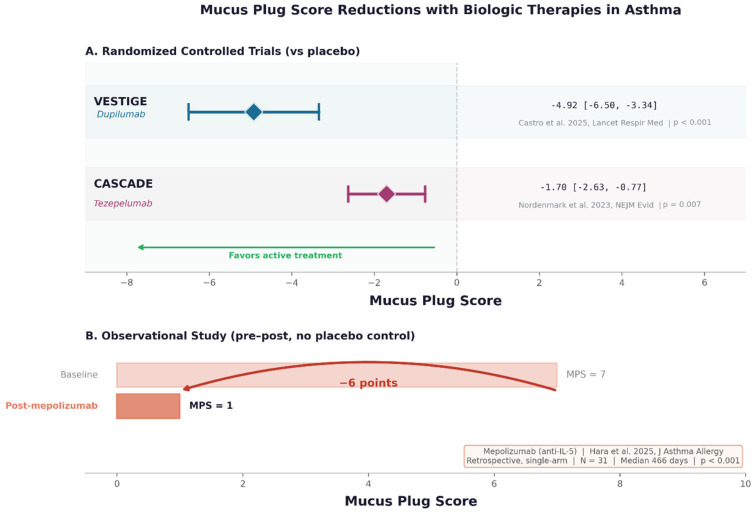
Summary of mucus plug score reductions in randomized controlled trials of biologics in asthma. Forest plot showing the change from baseline in mucus plug score for dupilumab (VESTIGE), tezepelumab (CASCADE) and mepolizumab (observational study) [12,13,34].

The systematic review by Aegerter and colleagues confirmed that, among T2-high populations, the mucus plug reduction was comparable across the biologics studied in RCTs, but residual plugs were observed in all studies after biologic intervention. Figure 3 provides an overview of the evidence for biologic-mediated mucus plug reduction across agents, including RCT data for dupilumab and tezepelumab and observational data for anti-IL-5/IL-5Rα agents. Residual plugs after treatment point to a gap in our knowledge: we still do not know enough about what plugs are made of, and how their composition affects the response to different biologics.

### 4.4. Omalizumab

Omalizumab (anti-IgE) has demonstrated mucus plug score reductions in single-arm observational studies [14]. Its mechanism of action on mucus is indirect, operating through reduction in IgE-mediated mast cell activation and downstream T2 cytokine release. Given the absence of RCT data, the magnitude and clinical significance of omalizumab’s effect on mucus plugs remain to be established. Table 1 summarizes the available evidence on biologic-mediated mucus plug reduction across agents and study designs.

## 5. Expanding the Paradigm to Eosinophilic COPD

The emergence of eosinophilic COPD as a distinct clinical endotype has been one of the most significant developments in respiratory medicine in recent years. T2 inflammation, as defined by blood eosinophil counts ≥ 300 cells/μL, is present in 20–40% of COPD patients and identifies a subgroup with increased exacerbation risk and potential responsiveness to biologic therapy [37].

The pooled analysis of BOREAS and NOTUS demonstrated that dupilumab, added to triple inhaled therapy, reduced the annualized rate of moderate or severe exacerbations by 31.3% (rate ratio 0.687, 95% CI 0.595–0.793; *p* < 0.0001) in eosinophilic COPD. The MATINEE trial showed a 21% reduction in exacerbation rates with mepolizumab (rate ratio 0.79, 95% CI 0.66–0.94; *p* = 0.01). An indirect comparison by Suter and colleagues suggested that dupilumab demonstrated statistically significant improvements in quality of life, symptoms, and FEV_1_ that were not observed with mepolizumab, particularly in patients with elevated FeNO (≥20 ppb) [38].

### Differential Trial Populations: Implications for Mucus Plug Biology

These two trial programs differ in their enrollment criteria. BOREAS and NOTUS required blood eosinophil counts ≥ 300 cells/μL during screening, but—and this is the key point—they also asked for a history of chronic productive cough for at least 3 months in the previous year, along with two or more moderate exacerbations or one severe exacerbation on triple therapy. MATINEE set a similar eosinophil threshold and exacerbation history but did not specify chronic productive cough and did not require FeNO assessment as a stratification factor.

What does this mean for the mucus plug discussion? The cough criterion in BOREAS/NOTUS may have enriched the study population for patients with a greater mucus burden—patients in whom plugging contributes more substantially to airflow obstruction. Dupilumab’s dual IL-4/IL-13 blockade would be expected to address both the eosinophilic inflammation and the IL-13-driven goblet cell metaplasia responsible for mucus hypersecretion. The superior FEV_1_ improvement observed with dupilumab versus mepolizumab at 12 weeks in the indirect comparison [38] could, at least in part, reflect a contribution of mucus plug resolution in a population pre-selected for mucus-related obstruction. This interpretation should be considered with caution, as the two trial programs differed in multiple design features beyond the cough criterion, and indirect comparisons carry inherent limitations. Mepolizumab, targeting IL-5 selectively, would not be expected to address this mechanism directly.

Furthermore, the greater efficacy signal in patients with elevated FeNO (≥20 ppb) observed with dupilumab in the BOREAS/NOTUS program aligns with the known association between high FeNO, IL-13-driven epithelial biology, and MUC5AC overexpression. Since FeNO reflects epithelial IL-13 signaling rather than eosinophil trafficking per se, it could potentially serve as a proxy biomarker, pending direct validation for the mucus-related component of T2 inflammation in COPD. This hypothesis warrants prospective testing through the inclusion of CT mucus plug scoring in future eosinophilic COPD trials.

None of the COPD trials, however, included mucus plug scoring as an endpoint. This represents a critical gap, given the accumulating evidence that mucus plugging occurs in COPD and contributes to airflow obstruction, disease progression, and mortality [39]. Oga and colleagues demonstrated that, unlike in asthma, elevated T2 biomarkers (FeNO ≥ 25 ppb and BEC ≥ 300 cells/μL) were not associated with mucus plugging in “pure” COPD, whereas patients with ACO exhibited mucus plug profiles similar to those seen in asthma [26]. This differential pattern suggests that the pathobiology of mucus plugging may differ between asthma and COPD even within the T2-high endotype, with implications for treatment selection.

Future trials of biologics in eosinophilic COPD should incorporate CT mucus plug scoring and, where feasible, FRI or MRI-based ventilation endpoints to characterize the impact of T2 cytokine blockade on mucus dynamics in this population.

## 6. Mucus Plugging as a Treatable Trait: A Framework for Clinical Practice

Agusti and colleagues proposed the treatable traits paradigm to move respiratory medicine beyond diagnostic labels toward the targeted treatment of specific, modifiable patient characteristics. Mucus plugging fits this framework. It is measurable through validated CT scoring, clinically relevant—associated with airflow obstruction, exacerbations, and impaired quality of life—and, as the trial evidence reviewed here demonstrates, modifiable through biologic therapy targeting T2 inflammation.

**Figure 4:**
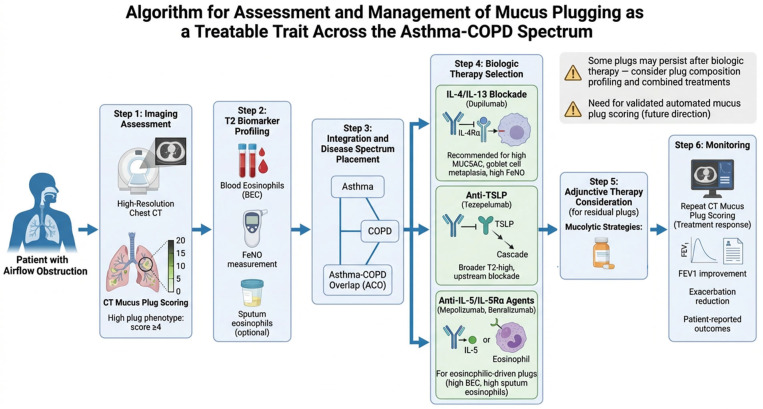
Proposed algorithm for the assessment and management of mucus plugging as a treatable trait across the asthma–COPD spectrum. The algorithm integrates CT mucus plug scoring, T2 biomarker profiling, and biologic therapy selection, including consideration of adjunctive mucolytic strategies for patients with residual mucus burden.

A practical approach to integrating mucus plug assessment into clinical decision-making can be broken down into three parts (Figure 4). The first is identification: routine CT imaging in patients with persistent airflow obstruction despite optimized inhaled therapy should include systematic evaluation for mucus plugs using a standardized scoring system. Second, characterization: correlation of mucus plug presence and score with T2 biomarkers (blood eosinophils, FeNO, sputum eosinophils where available) to determine the inflammatory driver. Third, targeted intervention: selection of the biologic agent most likely to address the dominant pathogenic mechanism—IL-4/IL-13 blockade (dupilumab) for patients with MUC5AC-driven mucus production and goblet cell metaplasia, anti-TSLP (tezepelumab) for patients with broader T2 activation, or anti-IL-5/IL-5Rα for patients with predominantly eosinophilic mucus plug composition.

What this amounts to, in practice, is treating the biology rather than the diagnosis [40]. In practical terms, mucus plug scoring could serve as a treatment-responsive biomarker for monitoring biologic efficacy—a complement to traditional endpoints like exacerbation rate, FEV_1_, and patient-reported outcomes that captures a dimension of disease currently invisible to spirometry alone.

## 7. Limitations, Mucolytic Strategies, and Future Directions

### 7.1. Limitations of the Current Evidence

Several limitations of the current evidence base can be noted. First, RCT data on mucus plug outcomes are limited to three trials in asthma [12,13,41], all with relatively small sample sizes (N = 82–109) and short follow-up periods (24–28 weeks). Whether these results apply to broader asthma populations and, critically, to COPD remains unestablished. Second, mucus plug scoring systems, while validated in the SARP cohort, rely on visual assessment by expert radiologists and lack standardized automated quantification; this makes scoring less consistent between readers and harder to use in large multicenter trials. Beyond standardization, several practical barriers limit the routine clinical adoption of CT-based mucus plug scoring. HRCT is not universally available in resource-limited settings, and repeated scanning raises concerns about cumulative radiation exposure, particularly in younger patients requiring longitudinal monitoring. The cost of HRCT, while modest relative to biologic therapy, adds to the overall economic burden of severe asthma management. Inter-reader variability in visual mucus plug scoring remains a concern, as the current scoring system relies on expert radiological assessment without universally adopted training protocols. AI-based automated quantification may mitigate some of these limitations by providing consistent, operator-independent measurements, but validation across diverse scanner platforms and patient populations is still needed. Third, the molecular composition of mucus plugs (eosinophilic vs. neutrophilic vs. mixed) cannot be determined from CT imaging alone, and how plug composition relates to treatment response remains unclear. Fourth, all biologic trials left behind some mucus plugs, suggesting that current drugs can slow new mucus production but cannot break down plugs that are already there.

### 7.2. Mucolytic Therapies: A Gap in the Evidence

An interesting gap in the current trial landscape is the lack of data on whether biologics and mucolytics work better together. While biologics target the upstream inflammatory drivers of mucus hypersecretion, established mucus plugs are composed of crosslinked mucin polymers, eosinophil-derived debris, DNA from extracellular traps, and crystalline galectin-10—components that require physical or biochemical disruption for clearance. N-acetylcysteine (NAC), a thiol-reducing agent, could theoretically disrupt the EPO-mediated disulfide crosslinks that stiffen the mucus gel [42,43]. Erdosteine and carbocisteine, which modulate mucin glycoprotein structure, may reduce mucus viscosity through complementary mechanisms. Dornase alfa, a recombinant DNase already established in cystic fibrosis, could target the DNA content of eosinophil extracellular traps within mucus plugs.

None of these agents have been evaluated in combination with biologics in patients with mucus-plugged airways, and none of the biologic trials in asthma or COPD have controlled for concomitant mucolytic use. This represents an unmet need of particular clinical relevance: patients with established, structurally complex mucus plugs may require a “dual-target” approach that combines anti-inflammatory biologics (to halt new mucus production) with mucolytic agents (to facilitate clearance of pre-existing plugs). The emerging anti-galectin-10 crystal-dissolving antibody approach represents a novel, mechanism-based strategy that could address the CLC component of eosinophilic mucus plugs but remains in preclinical development.

### 7.3. Where Do We Go from Here?

A number of questions remain, still, open:

(a) **Mucus plug scoring as a prespecified endpoint in COPD biologic trials.** Given the strong rationale for T2-driven mucus plugging in eosinophilic COPD and the differential FEV_1_ responses observed between dupilumab and mepolizumab, future phase 3 and phase 4 trials should incorporate CT mucus plug scoring as a secondary or exploratory endpoint. The inclusion of FeNO-stratified subgroup analyses for mucus outcomes would help delineate the IL-13-dependent component of plug formation in COPD.
(b) **Development and validation of automated AI-driven mucus plug detection.** Computer-based tools, once trained on labeled HRCT images, could count and locate mucus plugs automatically and consistently. Automated segmentation of plugged airways has been described in pilot work, but it still needs to be tested on different scanners and in different patient groups before it can be used widely. A notable step in this direction was taken by van der Veer and colleagues, who applied an AI-based automated quantification platform (LungQ) to chest CT scans of 9399 participants from the COPDGene study [44]. Automated mucus plug scores were significantly associated with all-cause mortality in participants with COPD GOLD stages 1–4, with adjusted hazard ratios of 1.18 (95% CI 1.05–1.32) for 1–2 obstructed segments and 1.27 (95% CI 1.10–1.46) for three or more obstructed segments—results closely mirroring those obtained by manual visual counting. This large-scale validation demonstrates that AI-based mucus plug quantification can reproduce expert-level prognostic stratification and is scalable for use in multicenter clinical trials and routine clinical practice.
(c) **Multimodal imaging integration.** Combining CT mucus plug scoring with functional endpoints—FRI-derived regional airway volumes, hyperpolarized gas MRI ventilation defect mapping, or parametric response mapping of small airway disease—would provide a comprehensive assessment of mucus plug burden and its functional consequences. Such multimodal approaches could enable more sensitive detection of treatment response than any single imaging modality alone.
(d) **Plug composition phenotyping and treatment prediction.** The development of non-invasive or minimally invasive methods to characterize mucus plug composition (eosinophilic vs. neutrophilic vs. mixed) is a key unmet need. CT attenuation characteristics, sputum proteomics, or exhaled biomarker profiles (FeNO, exhaled volatile organic compounds) may serve as proxies for plug composition and predict differential response to IL-4/IL-13 blockade versus anti-IL-5 versus mucolytic strategies.
(e) **Combination biologic + mucolytic trials.** Prospective randomized trials evaluating the addition of mucolytic agents (NAC, erdosteine, or inhaled hypertonic saline) to biologic therapy in patients with documented mucus plugging at baseline would address the critical question of whether accelerating plug clearance enhances the functional and clinical benefits of cytokine blockade. The optimal timing of mucolytic initiation (pre-biologic to clear baseline plugs, or concurrent) also warrants investigation.
(f) **Real-world and head-to-head studies.** Real-world studies evaluating mucus plug dynamics during biologic therapy, including head-to-head comparisons of different biologic classes with standardized imaging protocols, are needed to inform clinical decision-making [45,46]. Registries incorporating CT mucus plug data alongside clinical outcomes would enable long-term assessment of plug trajectory and its correlation with exacerbation risk and lung function decline.
(g) **Mucus plugging in the ACO zone.** The finding that ACO patients exhibit mucus plug profiles intermediate between asthma and COPD suggests that this population may represent an enriched target for biologic-mediated plug reduction. Dedicated studies in ACO are needed, given that this group is typically excluded from both asthma and COPD biologic trials.

## 8. Conclusions

Mucus plugging is emerging as a treatable trait of real clinical consequence across the asthma–COPD spectrum. Three developments have converged to make this possible: advanced quantitative imaging that renders plugs visible and measurable, a molecular understanding of how T2 cytokines drive their formation, and biologic drugs that can reduce them. Dupilumab and tezepelumab have shown meaningful reductions in CT-assessed mucus plug burden in asthma. Whether these agents achieve the same in eosinophilic COPD remains unknown—but the rationale is compelling. The divergent inclusion criteria of the BOREAS/NOTUS and MATINEE programs raise a thought-provoking hypothesis: that dupilumab’s superior lung function effects may partly reflect mucus plug resolution in a population pre-selected for mucus-related obstruction. If confirmed, this would change the way we choose which patients get biologic therapy. Integrating mucus plug assessment into the treatable traits framework, alongside adjunctive mucolytic strategies, would represent a real move away from empirical prescribing toward decisions guided by biology and imaging. Further studies are warranted to validate mucus plug scoring as a treatment-responsive biomarker in COPD, to develop automated imaging algorithms, and to evaluate combined biologic-plus-mucolytic regimens that address both the inflammatory cause and the physical makeup of pathologic airway mucus.

## Figures and Tables

**Table 1:** Summary of biologic therapy effects on CT-assessed mucus plug scores in asthma.

Study	Agent	Mechanism	Design	N	Follow-Up	Baseline MPS	Post-Tx MPS/Change	Key Outcomes
Nordenmark et al. 2023 [13]	Tezepelumab	Anti-TSLP	RCT (CASCADE)	82	28 wk	NR	Δ − 1.7 ± 2.6 vs. no change (placebo)	MPS reduction correlated with ↓ eosinophils, ↓ FeNO, ↑ FEV_1_
Castro et al. 2025 [12]	Dupilumab	Anti-IL-4Rα	RCT (VESTIGE)	109	24 wk	Mean ~ 6.0 (ITT)	Significant ↓ MPS and mucus volume vs. placebo	↑ siVaw (NS), ↓ FeNO, ↑ FEV_1_
Porsbjerg et al. 2025 [12]	Dupilumab	Anti-IL-4Rα	Post hoc (VESTIGE)	109	24 wk	High (≥4): 67.2%	High MPS: 67.2% → 32.8%	Pre-BD FEV_1_ LSMD + 16.77 pp (*p* < 0.0001) in high MPS; r = −0.61 ΔMPS–ΔFEV_1_
Hara et al. 2025 [34]	Mepolizumab	Anti-IL-5	Retrospective	31	~466 d	Median 7	Median 1 (*p* < 0.001)	ΔMPS correlated with ΔACT (r = −0.47) and ΔFEV_1_ (r = −0.68)
Campisi et al. 2025 [35]	Mepolizumab	Anti-IL-5	Prospective	47	12 mo	Median 4 (3–7)	Median 1 (0–2) (*p* < 0.0001)	ΔMPS correlated with Δsputum eos (r = 0.58), ΔFEV_1_ (r = −0.37)
Götschke et al. 2025 [36]	Multiple †	Mixed	Retrospective	113 (101 FU)	4 mo	Median 4; 77% with plugs	Not reassessed	Baseline MPS predicted ΔFEV_1_ (β = 0.72, *p* = 0.01) and ΔACT (β = 0.24, *p* = 0.001)

† Multiple agents: Anti-IgE 8.8%, Anti-IL-5 27.4%, Anti-IL-5R 37.2%, Anti-IL-4R 25.7%, Anti-TSLP 0.9%. Abbreviations: ACT, Asthma Control Test; BD, bronchodilator; eos, eosinophils; FU, follow-up; LSMD, least squares mean difference; MPS, mucus plug score; NR, not reported; NS, not significant; pp, percentage points; RCT, randomized controlled trial; siVaw, specific imaging airway volume; TSLP, thymic stromal lymphopoietin; Tx, treatment; wk, weeks.

## Data Availability

No new data were created or analyzed in this study. Data sharing is not applicable to this article.

## References

[1] Global Initiative for Chronic Obstructive Lung Disease (GOLD) (2024). Global Strategy for the Diagnosis, Management, and Prevention of COPD. http://www.Goldcopd.org.

[2] GINA Global Initiative for Asthma (GINA) (2024). Global Strategy for Asthma Management and Prevention. http://www.Ginasthma.org.

[3] Agusti A., Bel E., Thomas M., Vogelmeier C., Brusselle G., Holgate S., Humbert M., Jones P., Gibson P.G., Vestbo J. (2016). Treatable Traits: Toward Precision Medicine of Chronic Airway Diseases. Eur. Respir. J..

[4] Dunican E.M., Elicker B.M., Gierada D.S., Nagle S.K., Schiebler M.L., Newell J.D., Raymond W.W., Lachowicz-Scroggins M.E., Di Maio S., Hoffman E.A. (2018). Mucus Plugs in Patients with Asthma Linked to Eosinophilia and Airflow Obstruction. J. Clin. Investig..

[5] Ueki S., Dunican E., Diaz A.A. (2026). Mucus Plugs and Eosinophilic Inflammation: Expanding the Paradigm to COPD. J. Allergy Clin. Immunol. Pract..

[6] Tang M., Elicker B.M., Henry T., Gierada D.S., Schiebler M.L., Huang B.K., Peters M.C., Castro M., Hoffman E.A., Fain S.B. (2022). Mucus Plugs Persist in Asthma, and Changes in Mucus Plugs Associate with Changes in Airflow over Time. Am. J. Respir. Crit. Care Med..

[7] Nakagome K., Nagata M. (2024). The Possible Roles of IL-4/IL-13 in the Development of Eosinophil-Predominant Severe Asthma. Biomolecules.

[8] Aegerter H., Lambrecht B.N. (2023). The Pathology of Asthma: What Is Obstructing Our View?. Annu. Rev. Pathol..

[9] Tang M., Charbit A.R., Johansson M.W., Jarjour N.N., Denlinger L.C., Raymond W.W., Peters M.C., Dunican E.M., Castro M., Sumino K. (2024). Utility of Eosinophil Peroxidase as a Biomarker of Eosinophilic Inflammation in Asthma. J. Allergy Clin. Immunol..

[10] Persson E.K., Verstraete K., Heyndrickx I., Gevaert E., Aegerter H., Percier J.-M., Deswarte K., Verschueren K.H.G., Dansercoer A., Gras D. (2019). Protein Crystallization Promotes Type 2 Immunity and Is Reversible by Antibody Treatment. Science.

[11] Aegerter H., Brightling C.E., Dunican E.M., Lambrecht B.N., Lugogo N.L., Newell J.D., Porsbjerg C., Svenningsen S., Clarke D., Lindsley A.W. (2026). Effectiveness of Biologics for Reducing Occlusive Mucus Plugs in Patients with Severe Asthma: A Systematic Review. Respir. Res..

[12] Castro M., Papi A., Porsbjerg C., Lugogo N.L., Brightling C.E., González-Barcala F.-J., Bourdin A., Ostrovskyy M., Staevska M., Chou P.-C. (2025). Effect of Dupilumab on Exhaled Nitric Oxide, Mucus Plugs, and Functional Respiratory Imaging in Patients with Type 2 Asthma (VESTIGE): A Randomised, Double-Blind, Placebo-Controlled, Phase 4 Trial. Lancet Respir. Med..

[13] Nordenmark L.H., Hellqvist Å., Emson C., Diver S., Porsbjerg C., Griffiths J.M., Newell J.D., Peterson S., Pawlikowska B., Parnes J.R. (2023). Tezepelumab and Mucus Plugs in Patients with Moderate-to-Severe Asthma. NEJM Evid..

[14] Venegas Garrido C., Mukherjee M., Svenningsen S., Nair P. (2024). Eosinophil-Mucus Interplay in Severe Asthma: Implications for Treatment with Biologicals. Allergol. Int..

[15] Bhatt S.P., Rabe K.F., Hanania N.A., Vogelmeier C.F., Bafadhel M., Christenson S.A., Papi A., Singh D., Laws E., Dakin P. (2025). Dupilumab for Chronic Obstructive Pulmonary Disease with Type 2 Inflammation: A Pooled Analysis of Two Phase 3, Randomised, Double-Blind, Placebo-Controlled Trials. Lancet Respir. Med..

[16] Sciurba F.C., Criner G.J., Christenson S.A., Martinez F.J., Papi A., Roche N., Bourbeau J., Korn S., Bafadhel M., Han M.K. (2025). Mepolizumab to Prevent Exacerbations of COPD with an Eosinophilic Phenotype. N. Engl. J. Med..

[17] Choi W., Choe S., Lau G.W. (2020). Inactivation of FOXA2 by Respiratory Bacterial Pathogens and Dysregulation of Pulmonary Mucus Homeostasis. Front. Immunol..

[18] Lachowicz-Scroggins M.E., Finkbeiner W.E., Gordon E.D., Yuan S., Zlock L., Bhakta N.R., Woodruff P.G., Fahy J.V., Boushey H.A. (2017). Corticosteroid and Long-Acting ß-Agonist Therapy Reduces Epithelial Goblet Cell Metaplasia. Clin. Exp. Allergy.

[19] Bonser L.R., Erle D.J. (2017). Airway Mucus and Asthma: The Role of MUC5AC and MUC5B. J. Clin. Med..

[20] Bonser L.R., Zlock L., Finkbeiner W., Erle D.J. (2016). Epithelial Tethering of MUC5AC-Rich Mucus Impairs Mucociliary Transport in Asthma. J. Clin. Investig..

[21] Sheehan J.K., Kesimer M., Pickles R. (2006). Innate Immunity and Mucus Structure and Function. Novartis Found. Symp..

[22] Everman J.L., Sajuthi S.P., Liegeois M.A., Jackson N.D., Collet E.H., Peters M.C., Chioccioli M., Moore C.M., Patel B.B., Dyjack N. (2024). A Common Polymorphism in the Intelectin-1 Gene Influences Mucus Plugging in Severe Asthma. Nat. Commun..

[23] Corcoran T.E., Huber A.S., Hill S.L., Locke L.W., Weber L., Muthukrishnan A., Heidrich E.M., Wenzel S., Myerburg M.M. (2021). Mucociliary Clearance Differs in Mild Asthma by Levels of Type 2 Inflammation. Chest.

[24] Tanabe N., Matsumoto H. (2026). From Mucus Plugging to Airway Dilatation in Chronic Airway Diseases: A Perspective on the Contribution of the Airway Microbiome and Inflammation. Allergol. Int..

[25] Leung C., Tang M., Huang B.K., Fain S.B., Hoffman E.A., Choi J., Dunican E.M., Mauger D.T., Denlinger L.C., Jarjour N.N. (2024). A Novel Air Trapping Segment Score Identifies Opposing Effects of Obesity and Eosinophilia on Air Trapping in Asthma. Am. J. Respir. Crit. Care Med..

[26] Oga K., Hayashi Y., Tanabe N., Maetani T., Shiraishi Y., Sakamoto R., Sunadome H., Sato S., Matsumoto H., Sato A. (2025). Differential Associations between Type 2 Inflammation and Airway Mucus Plugs in Asthma, Asthma-COPD Overlap, and COPD. Respir. Investig..

[27] Mummy D.G., Dunican E.M., Carey K.J., Evans M.D., Elicker B.M., Newell J.D., Gierada D.S., Nagle S.K., Schiebler M.L., Sorkness R.L. (2022). Mucus Plugs in Asthma at CT Associated with Regional Ventilation Defects at ^3^He MRI. Radiology.

[28] Trivedi A.P., Hall C., Goss C.W., Lew D., Krings J.G., McGregor M.C., Samant M., Sieren J.P., Li H., Schechtman K.B. (2022). Quantitative CT Characteristics of Cluster Phenotypes in the Severe Asthma Research Program Cohorts. Radiology.

[29] Wang K., Ma D., Liu J., Zeng Y., Zhou Q., Zhang S., Jiang J., Lei W., Chen S., Chen G. (2025). Associations of Mucus Plugging, Small Airway Dysfunction, and Airway Wall Thickening in Cough-Variant Asthma versus Asthma. Eur. J. Med. Res..

[30] Colantuono S., Menzella F., Mari P.-V., Macagno F., Lombardi F., Baglivo I., Caruso C. (2025). Patient Response and Remission in Respiratory Disease: Special Focus on Severe Asthma and Chronic Obstructive Pulmonary Disease. J. Int. Med. Res..

[31] Carriera L., Mari P.V., Barone R., Ielo S., Cataldo E., Di Tomasso M., Bezzi M., Smargiassi A., Inchingolo R., Coppola A. (2025). Potential Novel Role of Asthma Biologics as Rescue Therapy in the Intensive Care Unit for Life-Threatening Asthma Exacerbations: A Systematic Review. Monaldi Arch. Chest Dis..

[32] Porsbjerg C., Dunican E.M., Lugogo N.L., Castro M., Papi A., Backer V., Brightling C.E., Bourdin A., Virchow J.C., Zhang M. (2026). Effect of Dupilumab on Mucus Burden in Patients with Moderate-to-Severe Asthma: The VESTIGE Trial. Am. J. Respir. Crit. Care Med..

[33] Fahy J.V. (2026). Progress in Treating Mucus Plugs in Asthma. Am. J. Respir. Crit. Care Med..

[34] Hara Y., Tanabe N., Marumo S., Murohashi K., Hayashi Y., Nagata M., Asada T., Nagaoka S., Kobayashi N., Hirai T. (2025). Clinical Impact of Mepolizumab on Airway Mucus Plugs in Patients with Severe Asthma. J. Asthma Allergy.

[35] Campisi R., Nolasco S., Bonsignore M., Nardo A.A., Intravaia R., Pelaia C., Vancheri C., Crimi N., Crimi C. (2025). Effectiveness of Mepolizumab on Mucus Plug Reduction and Clinical Outcomes in Severe Eosinophilic Asthma: A Prospective Observational Study. J. Allergy Clin. Immunol. Pract..

[36] Götschke J., Walter J., Leuschner G., Gerckens M., Götschke M., Mertsch P., Mümmler C., Lenoir A., Barnikel M., Dinkel J. (2025). Mucus Plug Score Predicts Clinical and Pulmonary Function Response to Biologic Therapy in Patients with Severe Asthma. J. Allergy Clin. Immunol. Pract..

[37] Singh D., Agusti A., Anzueto A., Barnes P.J., Bourbeau J., Celli B.R., Criner G.J., Frith P., Halpin D.M.G., Han M. (2019). Global Strategy for the Diagnosis, Management, and Prevention of Chronic Obstructive Lung Disease: The GOLD Science Committee Report 2019. Eur. Respir. J..

[38] Suter P., Greig R., Chan R., Lipworth B. (2025). Efficacy of Dupilumab and Mepolizumab in Eosinophilic COPD: Insights from Phase 3 Trials. Respir. Med..

[39] Krimsky W.S., Mammarappallil J.G., Kim V., Bannan B., Charbonnier J.-P., Hatton B.A., Sciurba F.C. (2026). Airway Mucus Plugging in Chronic Bronchitis and the Impact of Bronchial Rheoplasty. Chest.

[40] McDonald V.M., Fingleton J., Agusti A., Hiles S.A., Clark V.L., Holland A.E., Marks G.B., Bardin P.P., Beasley R., Pavord I.D. (2019). Treatable Traits: A New Paradigm for 21st Century Management of Chronic Airway Diseases: Treatable Traits Down Under International Workshop Report. Eur. Respir. J..

[41] Svenningsen S., Kjarsgaard M., Haider E., Venegas C., Konyer N., Friedlander Y., Engel R., Shen C., Nair P. (2023). Effects of Dupilumab on Mucus Plugging and Ventilation Defects in Patients with Moderate-to-Severe Asthma: A Randomized, Double-Blind, Placebo-Controlled Trial. Am. J. Respir. Crit. Care Med..

[42] Dal Negro R.W., Wedzicha J.A., Iversen M., Fontana G., Page C., Cicero A.F., Pozzi E., Calverley P.M.A. (2017). RESTORE group; RESTORE study. Effect of Erdosteine on the Rate and Duration of COPD Exacerbations: The RESTORE Study. Eur. Respir. J..

[43] Zheng J.-P., Wen F.-Q., Bai C.-X., Wan H.-Y., Kang J., Chen P., Yao W.-Z., Ma L.-J., Li X., Raiteri L. (2014). Twice Daily N-Acetylcysteine 600 Mg for Exacerbations of Chronic Obstructive Pulmonary Disease (PANTHEON): A Randomised, Double-Blind Placebo-Controlled Trial. Lancet Respir. Med..

[44] Van der Veer T., Andrinopoulou E.-R., Braunstahl G.-J., Charbonnier J.P., Kim V., Latisenko R., Lynch D.A., Tiddens H. (2025). Association between Automatic AI-Based Quantification of Airway-Occlusive Mucus Plugs and All-Cause Mortality in Patients with COPD. Thorax.

[45] Lipworth B.J., Greig R., Chan R., Kuo C.R., Jackson C. (2025). Head-To-Head Comparison of Biologic Efficacy in Asthma: What Have We Learned?. Allergy.

[46] Lavere P.F., Phillips K.M., Hanania N.A., Adrish M. (2025). Established and Emerging Asthma Biomarkers with a Focus on Biologic Trials: A Narrative Review. J. Pers. Med..

